# Serum semaphorin 7A is associated with the risk of acute atherothrombotic stroke

**DOI:** 10.1111/jcmm.14186

**Published:** 2019-02-07

**Authors:** Tao You, Zhengbao Zhu, Xiaowei Zheng, Nimei Zeng, Shuhong Hu, Yifei Liu, Lijie Ren, Qiongyu Lu, Chaojun Tang, Changgeng Ruan, Yonghong Zhang, Li Zhu

**Affiliations:** ^1^ Cyrus Tang Medical Institute, Collaborative Innovation Center of Hematology Soochow University Suzhou Jiangsu China; ^2^ Jiangsu Institute of Hematology, Key Laboratory of Thrombosis and Hemostasis of Ministry of Health The First Affiliated Hospital of Soochow University Suzhou Jiangsu China; ^3^ Department of Epidemiology, School of Public Health Medical College of Soochow University Suzhou China; ^4^ Jiangsu Key Laboratory of Preventive and Translational Medicine for Geriatric Diseases Soochow University Suzhou China; ^5^ State Key Laboratory of Radiation Medicine and Protection Soochow University Suzhou China

**Keywords:** acute atherothrombotic stroke, risk factor, semaphorin 7A

## Abstract

Semaphorin 7A (Sema7A), a neural guidance cue, was recently identified to regulate atherosclerosis in mice. However, the clinical relevance of Sema7A with atherosclerotic diseases remains unknown. The aim of this study was to investigate the association between serum Sema7A and the risk of acute atherothrombotic stroke (AAS). We measured serum concentrations of Sema7A in 105 newly onset AAS cases and 105 age‐ and sex‐matched controls, showing that median Sema7A level in AAS cases was over three times of that in controls (5.86 vs 1.66 ng/mL). Adjusted for hypertension, body mass index, fasting blood glucose, total cholesterol, triglyceride, high‐density lipoprotein (HDL)‐cholesterol, low‐density lipoprotein (LDL)‐cholesterol, current smoking and alcohol consumption, multivariate logistic regression showed that higher Sema7A was independently associated with the odds of AAS (OR = 6.40, 95% CI: 2.88‐14.25). Each 1‐standard deviation increase in Sema7A was associated with a threefold higher odds of AAS (OR = 3.42, 95% CI: 1.84‐6.35). Importantly, adding Sema7A to a multivariate logistic model containing conventional cardiovascular risk factors improved the area under receiver operating characteristic curves from 0.831 to 0.891 for the association with AAS. In conclusion, elevated serum Sema7A is independently associated with the risk of AAS, suggesting that it may play a potential role in AAS.

**List of main topics**

- Serum Sema7A is increased in patients with AAS.
- Higher serum Sema7A is independently associated with the risk of AAS.

## 1 INTRODUCTION

Stroke is one of the leading causes of mortality and disability worldwide. Acute atherothrombotic stroke (AAS), the most common subtype, is a result of the atherosclerosis of cerebral arteries accounting for an overwhelming majority of deaths in China.^1^, ^2^ The proatherogenic milieu consists of dysfunctional endothelium, activated leucocytes, platelets and inflammatory factors that facilitate atheroprogression and cardiovascular events.^3^, ^4^ These molecules, reflecting systemic or focal inflammation, have shed light on the risk stratification of AAS.^5^, ^6^ To date, emerging evidence suggests the roles of semaphorins, a protein family primarily detected on immunocyte membranes and signalling through plexins or integrins to exert versatile functions,^7^ underlying atherosclerotic diseases since their initial demonstration as axonal guidance cues.^8^, ^9^, ^10^ Among this family, semaphorin 7A (Sema7A) is a membrane protein encoded by *SEMA7A* gene on human chromosome 15 (25 kb) or mouse chromosome 9B (22 kb) and shows an important role in immune response, neural development, bone homeostasis, cancer and inflammation.^11^, ^12^, ^13^, ^14^ Up‐regulation of Sema7A expression has been associated with various inflammatory diseases, including interstitial lung disease,^15^ multiple sclerosis (MS),^16^ rheumatoid arthritis^17^ and airway hypersensitivity.^18^ Recently, we demonstrated a causal role of Sema7A in experimental atherosclerosis via eliciting endothelial dysfunction and vascular inflammation,^19^ whereas the clinical relevance of Sema7A in atherosclerotic disease remains to be elucidated. Therefore, we conducted this case‐control study to investigate the association of serum Sema7A with AAS.

## 2 MATERIALS AND METHODS

### 2.1 Study population

We randomly selected 105 cases from a large cohort of patients with AAS from CATIS (China Antihypertensive Trial in Acute Ischemic Stroke).^20^ In CATIS, patients with a systolic BP ≥220 mm Hg or diastolic BP ≥120 mm Hg, severe heart failure, acute myocardial infarction or unstable angina, atrial fibrillation, aortic dissection, cerebrovascular stenosis or resistant hypertension; those in a deep coma and those treated with intravenous thrombolytic therapy were excluded. The randomization process was conducted as follows: There were a total of 3170 patients with atherothrombotic stroke and with serum samples in CATIS. Among all these atherothrombotic stroke patients, we selected 105 patients as cases by means of simple random sampling method. Controls free of stroke from a cross‐sectional study in China were matched to the cases by 1:1 based on age (birth at the same year) and sex by SAS software.^21^ Subjects with conditions known to affect Sema7A levels, including rheumatoid arthritis, interstitial lung disease, idiopathic pulmonary fibrosis (IPF), polycystic kidney disease, alcoholic liver disease, MS and bone fracture were excluded. The protocol of this case‐control study conforms to the ethical guidelines of the Declaration of Helsinki^22^ and has been approved by the institutional review boards and ethics committees at Soochow University in China and all participating hospitals. Written informed consent was obtained from all study participants included in the study or their immediate family members.

### 2.2 Data collection and measurements

Baseline information on demographic characteristics, lifestyle risk factors, medical history and use of medications were collected at admission or recruitment by trained staffs using a standard questionnaire. Hypertension was defined as systolic blood pressure (SBP) ≥130 mm Hg and/or diastolic blood pressure (DBP) ≥80 mm Hg or currently under antihypertensive therapy.^23^ Body weight and height were measured with a routinely calibrated stadiometer and scale. Body mass index (BMI) was calculated as body weight (kilograms) divided by height (metres) squared. In this study, blood samples were collected for all participants in the morning after 8 hours of fasting. The incubation time for blood coagulation lasted for 4 hours at 4°C and then all serum samples were separated and frozen at −80°C until laboratory testing. Serum Sema7A concentrations were assessed centrally at the School of Public Health at Soochow University on a FilterMax F5 Multi‐Mode Microplate Reader using a commercially available enzyme‐linked immunosorbent assay kit (ELH‐SEMA7A, RayBiotech, Peachtree Corner, GA, USA) according to the manufacturer's instructions. The concentrations of Sema7A were determined according to a standard curve built for the same test set. Laboratory technicians who performed the measurements were blind to the cases and controls.

### 2.3 Statistical analysis

Continuous variables with normal and skewed distribution were presented as means with standard deviation (SD) and medians with interquartile ranges (IQRs) respectively. Means and medians in variables between the two groups were compared using the paired Student's *t* test or Wilcoxon rank‐sum test. Comparisons of percentages for categorical variables between the two groups were performed using the *χ* ^2^ test. Differences in Sema7A levels were evaluated between AAS cases and controls. Median Sema7A level (3.20 ng/mL) was served as cut‐off and the study population was divided into low Sema7A (<3.20 ng/mL) and high Sema7A (≥3.20 ng/mL) groups. Both univariate and multivariate logistic regression analyses were conducted to calculate the odds ratios (ORs) and 95% confidence intervals (95% CIs) of AAS associated with high Sema7A levels. In multivariate logistic analyses, adjustment variables included hypertension, BMI, current smoking, alcohol consumption, FBG, total cholesterol (TC), triglyceride (TG), low‐density lipoprotein cholesterol (LDL‐C) and high‐density lipoprotein cholesterol (HDL‐C). In addition, we assessed the improvement value of Sema7A for prediction of AAS risk by computing the area under receiver operating characteristic curves (AUC) and compared the model including Sema7A and other conventional risk factors to a model including only other conventional risk factors.^24^ The association between serum Sema7A and high‐sensitivity C‐reactive protein (hsCRP) was evaluated using linear correlation and regression. A two‐tailed *P* < 0.05 was considered statistically significant. Statistical analyses were performed using SPSS software (version 22.0, IBM, Armonk, NY, USA) and MedCalc (version 15, MedCalc Software, Ostend, Belgium).

## 3 RESULTS

**Figure 1:**
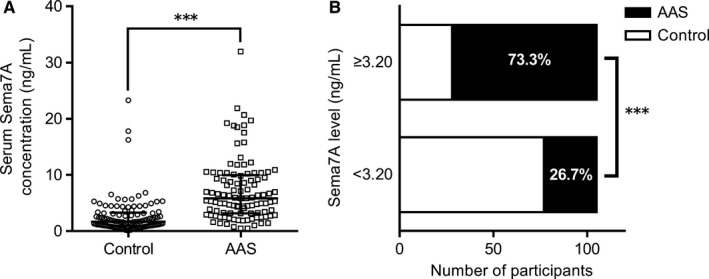
Comparison of Serum Sema7A levels between acute atherothrombotic stroke (AAS) patients and controls. (A) The scatter plot of Sema7A concentrations in the study population. Horizontal lines indicate medians and interquartile ranges. (B) Proportions of AAS cases in participants with higher and lower levels of Sema7A. AAS, acute atherothrombotic stroke. (***P < 0.001)

A total of 105 AAS patients and 105 age‐ and sex‐matched controls were included in the analyses. As shown in Table 1, AAS cases are more likely to have higher BMI, SBP, DBP, TG and Sema7A levels compared with controls. Serum Sema7A level in AAS patients was more than three times of that in controls (5.86 ng/mL [IQR 3.17‐9.97] vs 1.66 ng/mL [IQR 0.92‐3.29], *P < *0.001) (Figure 1A). After being divided by the median concentration of Sema7A (3.20 ng/mL), the proportion of cases was significantly higher in subjects with high Sema7A levels than that in those with low Sema7A levels (73.3% vs 26.7%, *P < *0.001) (Figure 1B). Further analyses in 98 AAS patients showed no significant association between serum Sema7A and hsCRP levels (Pearson Correlation: −0.119, *R* ^2^ = 0.014, *P* = 0.245) (Figure S1).

**Table 1:** Baseline characteristics of 105 acute atherothrombotic stroke patients and 105 age‐ and sex‐matched controls

Variable	Patients (n = 105)	Control (n = 105)	*P* value
Age (y)	54 (52‐58)	54 (52‐58)	0.691
Male, no. (%)	70 (66.7)	70 (66.7)	1.000
BMI (kg/m^2^)^a^	25.61 ± 2.58	24.28 ± 3.08	0.001
Systolic BP (mm Hg)^b^	162.0 (150.7‐179.7)	120.0 (111.0‐126.0)	<0.001
Diastolic BP (mm Hg)^b^	99.3 (91.3‐104.0)	79.0 (72.5‐83.0)	<0.001
Current smoking, no. (%)	52 (49.5)	54 (51.4)	0.783
Alcohol consumption, no. (%)	39 (37.1)	40 (38.1)	0.887
TC (mmol/L)^b^	5.09 (4.30‐5.82)	5.02 (4.41‐5.75)	0.960
TG (mmol/L)^b^	1.59 (1.06‐2.48)	1.19 (0.86‐1.94)	0.005
LDL‐C (mmol/L)^b^	2.95 (2.23‐3.52)	3.13 (2.59‐3.65)	0.087
HDL‐C (mmol/L)^b^	1.13 (0.97‐1.53)	1.18 (1.04‐1.39)	0.621
FBG (mmol/L)^b^	5.60 (5.00‐6.35)	5.47 (5.11‐6.08)	0.620
Sema7A (ng/mL)^b^	5.86 (3.17‐9.97)	1.66 (0.92‐3.29)	<0.001

BP, blood pressure; BMI, body mass index; TC, total cholesterol; TG, triglycerides; LDL‐C, low‐density lipoprotein cholesterol; HDL‐C, high‐density lipoprotein cholesterol; FBG, fasting blood glucose.

a Mean ± SD or

b median (interquartile range).

After controlling for conventional cardiovascular disease (CVD) risk factors including hypertension, BMI, FBG, TC, TG, HDL‐C, LDL‐C, current smoking and alcohol consumption, the multivariate‐adjusted OR (95% CI) of AAS for high Sema7A levels was 6.40 (2.88‐14.25) (*P < *0.001), compared with low Sema7A. Additionally, each 1‐SD increase of Sema7A was associated with a three times higher odds of AAS (adjusted OR = 3.42, 95% CI: 1.84‐6.35; *P < *0.001) (Table 2).

**Table 2:** Odds ratios and 95% confidence intervals of acute atherothrombotic stroke risk associated with serum Sema7A

	Number of participants	Unadjusted		Multivariate‐adjusted^a^	
		OR (95% CI)	*P* value	OR (95% CI)	*P* value
Sema7A, ng/mL					
<3.20	105	1.00 (reference)		1.00 (reference)	
≥3.20	105	7.56 (4.10‐13.94)	<0.001	6.40 (2.88‐14.25)	<0.001
Per SD increase		5.25 (2.93‐9.38)	<0.001	3.42 (1.84‐6.35)	<0.001

OR, odds ratio; SD, standard deviation; CI, confidence interval.

a Adjusted for hypertension, body mass index, total cholesterol, triglycerides, low‐density lipoprotein cholesterol, high‐density lipoprotein cholesterol, fasting blood glucose, current smoking and alcohol consumption.

**Figure 2:**
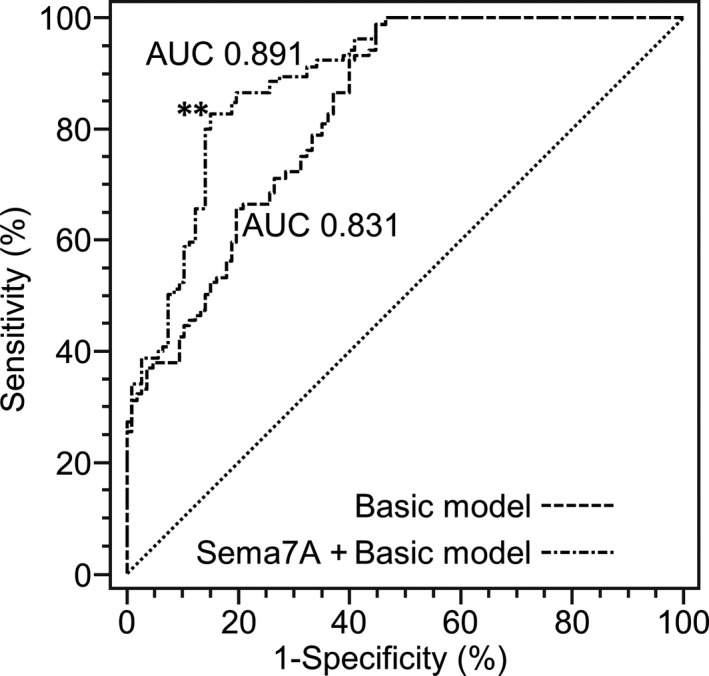
Receiver operating characteristic curves (ROC) of the logistic model including Sema7A and other conventional risk factors, and the model including only other conventional risk factors. Basic model: hypertension, body mass index, total cholesterol, triglycerides, low‐density lipoprotein cholesterol, high‐density lipoprotein cholesterol, fasting blood glucose, current smoking and alcohol consumption. AUC, area under curve. (**P < 0.01)

As displayed in Figure 2, the AUC for the model containing Sema7A and other conventional risk factors, including hypertension, BMI, FBG, TC, TG, HDL‐C, LDL‐C, current smoking and alcohol consumption, was significantly larger than that for the model including only other conventional risk factors (0.891 vs 0.831, *P* < 0.01), suggesting that incorporating Sema7A into the basic model significantly improved the prediction value of the model for AAS cases.

## 4 DISCUSSION

In this 1:1 matched case‐control study, we found that serum Sema7A level was significantly higher in AAS patients than that in healthy controls. Furthermore, in multivariate logistic analyses, increased serum Sema7A was significantly associated with the risk of AAS while controlling some important conventional CVD risk factors including hypertension, BMI, FBG, TC, TG, HDL‐C, LDL‐C, current smoking and alcohol consumption. In addition, adding Sema7A in a model containing the conventional CVD risk factors further improved the ability to distinguish AAS cases from controls beyond these factors. These findings suggest that Sema7A plays an important role in cerebral atherosclerosis.

To the best of our knowledge, the association between Sema7A and atherosclerotic diseases in human population has not yet been reported, despite a growing body of evidence on the role of Sema7A in several autoimmune and inflammatory disorders.^15^, ^16^, ^17^, ^18^, ^25^, ^26^, ^27^ For instance, sema7A was found to bind autoantibodies in synovial fluids from patients with rheumatoid arthritis.^26^ MS patients showed a positive correlation of astrocyte Sema7A with increased inflammatory activity.^16^ Moreover, Sema7A expression was up‐regulated in peripheral blood mononuclear cells (PBMCs)^15^ and T cells^25^ from patients with scleroderma‐related interstitial lung disease and IPF, and higher levels of Sema7A were observed in circulating regulatory T cells from patients with autosomal‐dominant polycystic kidney disease.^27^ Of note, these immunocytes are essential contributors to vascular inflammation, suggesting a potential link between Sema7A and atherosclerosis. In our recent study, we found that Sema7A expression was up‐regulated in an experimental mouse atherosclerosis model. Targeted Sema7A gene knockout protected mice from high‐fat diet and disturbed blood flow‐induced atherosclerosis. Compared with wild‐type littermates, *Sema7A* ^−/−^ mice had attenuated endothelial inflammation, leucocyte infiltration and plaque formation.^19^ To extend our understanding of Sema7A in atherosclerotic diseases, we selected AAS patients and a control population strictly matched for age and sex to investigate the clinical relevance of Sema7A. Before the current study, most of the human studies on Sema7A involved only a very small study population and lacked an appropriate control group. Moreover, previous studies used only simple statistical tests to explore the association between Sema7A and diseases without controlling for potential confounders.^15^, ^17^, ^25^, ^27^ In contrast, the stringent criteria for selecting cases and controls in our study ensure comparability between AAS and control groups and minimize the interference from potential confounders. We further adjusted for some potential confounders in multivariate logistic analyses. We demonstrated an important result that elevated Sema7A was highly associated with AAS, which provided strong support to our previous findings in animal models of atherosclerosis.

The mechanisms underlying the observed association of serum Sema7A with AAS have been proposed in our previous study^19^ and other studies. It is well documented that Sema7A and its receptors are expressed in leucocytes, endothelial cells (ECs), neurons and platelets,^28^, ^29^, ^30^ all of which play essential roles in atherosclerosis. The increased Sema7A observed in AAS may be originated from injured EC,^19^ activated leucocytes,^31^ platelets and erythrocytes.^28^, ^30^ Furthermore, enhanced enzymatic cleavage from cell surface under inflammatory conditions may lead to elevated serum soluble Sema7A levels.^17^, ^32^ Importantly, our recent study in mouse models of atherosclerosis demonstrated that increased EC Sema7A expression in endothelial dysfunction propagates vascular inflammation and atherosclerotic lesions.^19^ Also, Sema7A may contribute to the progression of atherosclerosis via directly inducing leucocyte activation and transmigration.^11^, ^33^ These results suggest that Sema7A may play an important role in the development of atherosclerosis. Synthetically considering the atheroprotection induced by Sema7A gene knockout in animal models^19^ and the observed association between Sema7A and AAS, we speculated that serum Sema7A had a strong association with atherosclerosis or stroke and might be a biomarker of atherosclerosis and therapeutic target in atherosclerotic diseases.

Considering potential limitations of this study, our results should be interpreted cautiously. The current study is a case‐control study with a relatively small study population, and with the nature of the cross section for the association of Sema7A with AAS because measurements of Sema7A were only obtained after AAS onset. Therefore, the association of elevated Sema7A with AAS needs to be validated in future prospective studies. This case‐control study aimed to investigate the association between Sema7A and AAS, while the association between other inflammatory factors and AAS was not evaluated, thus we were not able to compare the Sema7A level with other inflammation biomarkers in the whole study population and control their effects in the analyses. Since plasma Sema7A was not measured in this study, we were not able to compare the levels of Sema7A between serum and plasma. Finally, our study was conducted only in Chinese Han population, thus may limit the generalization of the findings to other ethnic groups.

In summary, our results demonstrated that increased serum Sema7A was independently associated with the risk of AAS, showing that Sema7A may play an important role in atherosclerosis. Further large‐scale prospective studies are warranted to verify our findings and determine the prognostic value of Sema7A in various atherosclerotic diseases.

## CONFLICT OF INTEREST

The authors confirm that there are no conflicts of interest.

## AUTHOR CONTRIBUTION

TY, ZZ, CT, CR, YZ and LZ designed the study. TY, ZZ, XZ, NZ, SH, YL, YZ and LZ performed the research. YZ and LZ contributed essential reagents. TY, ZZ, QL, LR, YZ and LZ analysed the data. TY, ZZ, YZ and LZ wrote and revised the paper.

## References

[1] **Roth** GA , **Johnson** C , **Abajobir** A , et al. Global, regional, and national burden of cardiovascular diseases for 10 causes, 1990 to 2015. J Am Coll Cardiol. 2017;70:1‐25. 28527533 10.1016/j.jacc.2017.04.052 PMC5491406

[2] **Wang** W , **Jiang** B , **Sun** H , et al. Prevalence, Incidence, and mortality of stroke in China: results from a nationwide population‐based survey of 480 687 adults. Circulation. 2017;135:759‐771. 28052979 10.1161/CIRCULATIONAHA.116.025250

[3] **Gerdes** N , **Seijkens** T , **Lievens** D , et al. Platelet CD40 exacerbates atherosclerosis by transcellular activation of endothelial cells and leukocytes. Arterioscler Thromb Vasc Biol. 2016;36:482‐490. 26821950 10.1161/ATVBAHA.115.307074

[4] **Back** M , **Hansson** GK . Anti‐inflammatory therapies for atherosclerosis. Nat Rev Cardiol. 2015;12:199‐211. 25666404 10.1038/nrcardio.2015.5

[5] **Pikula** A , **Beiser** AS , **DeCarli** C , et al. Multiple biomarkers and risk of clinical and subclinical vascular brain injury: the Framingham Offspring Study. Circulation. 2012;125:2100‐2107. 22456473 10.1161/CIRCULATIONAHA.110.989145 PMC3427730

[6] **Yeboah** J , **Young** R , **McClelland** RL , et al. Utility of nontraditional risk markers in atherosclerotic cardiovascular disease risk assessment. J Am Coll Cardiol. 2016;67:139‐147. 26791059 10.1016/j.jacc.2015.10.058 PMC4724058

[7] **Kruger** RP , **Aurandt** J , **Guan** KL . Semaphorins command cells to move. Nat Rev Mol Cell Biol. 2005;6:789‐800. 16314868 10.1038/nrm1740

[8] **Wanschel** A , **Seibert** T , **Hewing** B , et al. Neuroimmune guidance cue Semaphorin 3E is expressed in atherosclerotic plaques and regulates macrophage retention. Arterioscler Thromb Vasc Biol. 2013;33:886‐893. 23430613 10.1161/ATVBAHA.112.300941 PMC3647027

[9] **Zhu** L , **Stalker** TJ , **Fong** KP , et al. Disruption of SEMA4D ameliorates platelet hypersensitivity in dyslipidemia and confers protection against the development of atherosclerosis. Arterioscler Thromb Vasc Biol. 2009;29:1039‐1045. 19390055 10.1161/ATVBAHA.109.185405 PMC2877695

[10] van **Gils** JM , **Ramkhelawon** B , **Fernandes** L , et al. Endothelial expression of guidance cues in vessel wall homeostasis dysregulation under proatherosclerotic conditions. Arterioscler Thromb Vasc Biol. 2013;33:911‐919. 23430612 10.1161/ATVBAHA.112.301155 PMC3647028

[11] **Suzuki** K , **Okuno** T , **Yamamoto** M , et al. Semaphorin 7A initiates T‐cell‐mediated inflammatory responses through alpha1beta1 integrin. Nature. 2007;446:680‐684. 17377534 10.1038/nature05652

[12] **Pasterkamp** RJ , **Peschon** JJ , **Spriggs** MK , **Kolodkin** AL . Semaphorin 7A promotes axon outgrowth through integrins and MAPKs. Nature. 2003;424:398‐405. 12879062 10.1038/nature01790

[13] **Delorme** G , **Saltel** F , **Bonnelye** E , et al. Expression and function of semaphorin 7A in bone cells. Biol Cell. 2005;97:589‐597. 15859945 10.1042/BC20040103

[14] **Black** SA , **Nelson** AC , **Gurule** NJ , et al. Semaphorin 7a exerts pleiotropic effects to promote breast tumor progression. Oncogene. 2016;35:5170‐5178. 27065336 10.1038/onc.2016.49 PMC5720143

[15] **Gan** Y , **Reilkoff** R , **Peng** X , et al. Role of semaphorin 7a signaling in transforming growth factor beta1‐induced lung fibrosis and scleroderma‐related interstitial lung disease. Arthritis Rheum. 2011;63:2484‐2494. 21484765 10.1002/art.30386 PMC3651701

[16] **Costa** C , **Martinez‐Saez** E , **Gutierrez‐Franco** A , et al. Expression of semaphorin 3A, semaphorin 7A and their receptors in multiple sclerosis lesions. Mult Scler. 2015;21:1632‐1643. 26432853 10.1177/1352458515599848

[17] **Xie** J , **Wang** H . Semaphorin 7A as a potential immune regulator and promising therapeutic target in rheumatoid arthritis. Arthritis Res Ther. 2017;19:10. 28109308 10.1186/s13075-016-1217-5 PMC5251212

[18] **Esnault** S , **Kelly** EA , **Johansson** MW , et al. Semaphorin 7A is expressed on airway eosinophils and upregulated by IL‐5 family cytokines. Clin Immunol. 2014;150:90‐100. 24333536 10.1016/j.clim.2013.11.009 PMC3947215

[19] **Hu** S , **Liu** Y , **You** T , et al. Vascular Semaphorin 7A upregulation by disturbed flow promotes atherosclerosis through endothelial beta1 Integrin. Arterioscler Thromb Vasc Biol. 2018;38:335‐343. 29269512 10.1161/ATVBAHA.117.310491 PMC5785426

[20] **He** J , **Zhang** Y , **Xu** T , et al. Effects of immediate blood pressure reduction on death and major disability in patients with acute ischemic stroke: the CATIS randomized clinical trial. JAMA. 2014;311:479‐489. 24240777 10.1001/jama.2013.282543

[21] **Zhu** Z , **Zhang** Q , **Peng** H , et al. Predictive value of serum soluble corin in the risk of hyperglycemia: a population‐based prospective cohort study in China. Clin Chim Acta. 2018;479:138‐143. 29366831 10.1016/j.cca.2018.01.028

[22] **Shephard** DA . The 1975 declaration of Helsinki and consent. Can Med Assoc J. 1976;115:1191‐1192. 1000449 PMC1878977

[23] **Whelton** PK , **Carey** RM , **Aronow** WS , et al. 2017 ACC/AHA/AAPA/ABC/ACPM/AGS/APhA/ASH/ASPC/NMA/PCNA guideline for the prevention, detection, evaluation, and management of high blood pressure in adults: a report of the American college of cardiology/American heart association task force on clinical practice guidelines. J Am Coll Cardiol. 2018;71:e127‐e248. 29146535 10.1016/j.jacc.2017.11.006

[24] **DeLong** ER , **DeLong** DM , **Clarke‐Pearson** DL . Comparing the areas under two or more correlated receiver operating characteristic curves: a nonparametric approach. Biometrics. 1988;44:837‐845. 3203132

[25] **Reilkoff** RA , **Peng** H , **Murray** LA , et al. Semaphorin 7a+ regulatory T cells are associated with progressive idiopathic pulmonary fibrosis and are implicated in transforming growth factor‐beta1‐induced pulmonary fibrosis. Am J Respir Crit Care Med. 2013;187:180‐188. 23220917 10.1164/rccm.201206-1109OC PMC3570653

[26] **Kim** CW , **Cho** EH , **Lee** YJ , et al. Disease‐specific proteins from rheumatoid arthritis patients. J Korean Med Sci. 2006;21:478‐484. 16778393 10.3346/jkms.2006.21.3.478 PMC2729955

[27] **Lee** Y , **Blount** KL , **Dai** F , et al. Semaphorin 7A in circulating regulatory T cells is increased in autosomal‐dominant polycystic kidney disease and decreases with tolvaptan treatment. Clin Exp Nephrol. 2018;22:906‐916. 29453607 10.1007/s10157-018-1542-x

[28] **Fong** KP , **Barry** C , **Tran** AN , et al. Deciphering the human platelet sheddome. Blood. 2011;117:e15‐26. 20962327 10.1182/blood-2010-05-283838 PMC3037762

[29] **Jongbloets** BC , **Ramakers** GM , **Pasterkamp** RJ . Semaphorin7A and its receptors: pleiotropic regulators of immune cell function, bone homeostasis, and neural development. Semin Cell Dev Biol. 2013;24:129‐138. 23333497 10.1016/j.semcdb.2013.01.002

[30] **Angelisova** P , **Drbal** K , **Cerny** J , et al. Characterization of the human leukocyte GPI‐anchored glycoprotein CDw108 and its relation to other similar molecules. Immunobiology. 1999;200:234‐245. 10416131 10.1016/s0171-2985(99)80073-4

[31] **Suzuki** K , **Kumanogoh** A , **Kikutani** H . Semaphorins and their receptors in immune cell interactions. Nat Immunol. 2008;9:17‐23. 18087252 10.1038/ni1553

[32] **Menghini** R , **Fiorentino** L , **Casagrande** V , et al. The role of ADAM17 in metabolic inflammation. Atherosclerosis. 2013;228:12‐17. 23384719 10.1016/j.atherosclerosis.2013.01.024

[33] **Morote‐Garcia** JC , **Napiwotzky** D , **Kohler** D , **Rosenberger** P . Endothelial Semaphorin 7A promotes neutrophil migration during hypoxia. Proc Natl Acad Sci USA. 2012;109:14146‐14151. 22891341 10.1073/pnas.1202165109 PMC3435204

