# Supplementary figures and images for: Serum semaphorin 7A is associated with the risk of acute atherothrombotic stroke
Source: J Cell Mol Med. 2019 Feb 7;23(4):2901–6. doi: 10.1111/jcmm.14186 (PMC6433662; doi:10.1111/jcmm.14186)

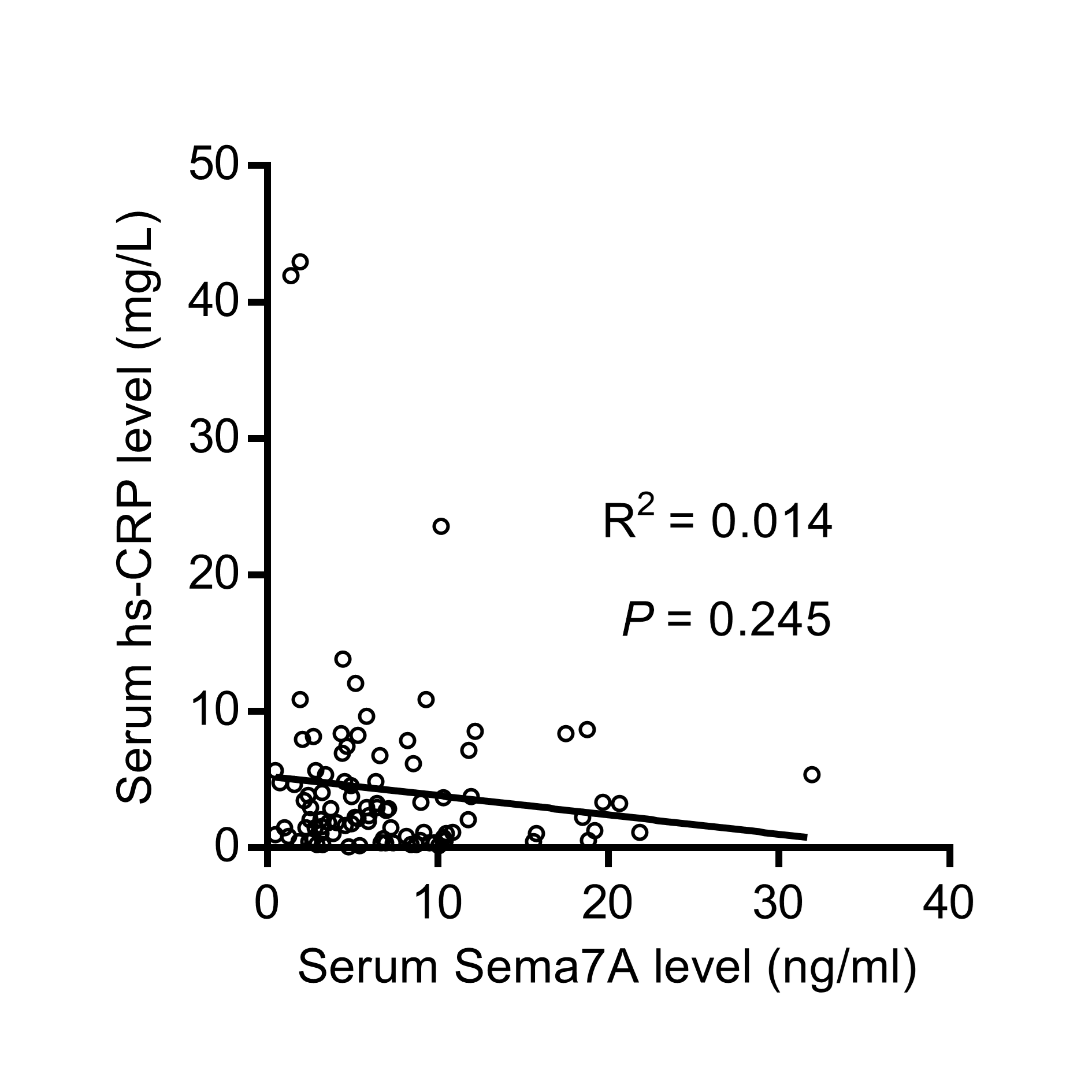

Supplement: Supplementary file 1 [file JCMM-23-2901-s001.tif]
